# Structure and Morphology of Crystalline Syndiotactic Polypropylene-Polyethylene Block Copolymers

**DOI:** 10.3390/polym14081534

**Published:** 2022-04-10

**Authors:** Rocco Di Girolamo, Alessandra Cicolella, Giovanni Talarico, Miriam Scoti, Fabio De Stefano, Angelo Giordano, Anna Malafronte, Claudio De Rosa

**Affiliations:** Dipartimento di Scienze Chimiche, Università degli Studi di Napoli Federico II, Complesso Monte S. Angelo, Via Cintia, 80126 Napoli, Italy; alessandra.cicolella@unina.it (A.C.); talarico@unina.it (G.T.); miriam.scoti@unina.it (M.S.); fabio.destefano@unina.it (F.D.S.); angelo.giordano@unina.it (A.G.); anna.malafronte@unina.it (A.M.)

**Keywords:** semicrystalline block copolymers, phase separation and crystallization, morphology, small-angle X-ray scattering

## Abstract

A study of the structure and morphology of diblock copolymers composed of crystallizable blocks of polyethylene (PE) and syndiotactic polypropylene (sPP) having different lengths is reported. In both analyzed samples, the PE block crystallizes first by cooling from the melt (at 130 °C) and the sPP block crystallizes after at a lower temperature. Small angle X-ray scattering (SAXS) recorded during cooling showed three correlation peaks at values of the scattering vector, *q*_1_ = 0.12 nm^−1^, *q*_2_ = 0.24 nm^−1^ and *q*_3_ = 0.4 nm^−1^, indicating development of a lamellar morphology, where lamellar domains of PE and sPP alternate, each domain containing stacks of crystalline lamellae of PE or sPP sandwiched by their own amorphous phase of PE or sPP. At temperatures higher than 120 °C, when only PE crystals are formed, the morphology is defined by the formation of stacks of PE lamellae (17 nm thick) alternating with amorphous layers and with a long period of nearly 52 nm. At lower temperatures, when crystals of sPP are also well-formed, the morphology is more complex. A model of the morphology at room temperature is proposed based on the correlation distances determined from the self-correlation functions extracted from the SAXS data. Lamellar domains of PE (41.5 nm thick) and sPP (8.2 nm thick) alternate, each domain containing stacks of crystalline lamellae sandwiched by their own amorphous phase, forming a global morphology having a total lamellar periodicity of 49.7 nm, characterized by alternating amorphous and crystalline layers, where the crystalline layers are alternatively made of stacks of PE lamellae (22 nm thick) and thinner sPP lamellae (only 3.5 nm thick).

## 1. Introduction

The structure and the morphology that develop in semicrystalline block copolymers (BCPs) depend on the competition between phase separation of the incompatible blocks and crystallization of one or more blocks [1,2,3,4,5,6,7,8]. Phase separation favors the formation of nanometer-sized microdomains whose shape, form and size depend on the BCP composition [9], and crystallization that favors the formation of alternating crystalline and amorphous layers [1,3,7,10,11]. The result of this competition is the possible formation of many different structures and morphologies at room temperature that depend on the crystallization and glass transition temperatures of blocks and the order–disorder transition temperature, and on which process between crystallization and phase separation occurs first upon cooling from the melt [1,2,3,4,5,6,7,8,9,10,11].

When the two polymer blocks are miscible in the melt, or weakly segregated, crystallization occurs from a homogeneous melt driving microphase separation and the final structure is essentially defined by the crystal morphology [7,10,11]. When the two blocks are strongly segregated in the melt, crystallization occurs from a microphase-separated heterogeneous melt, resulting in crystallization confined within microdomains formed in the melt by phase separation, templated crystallization or breaking out of the nanostructure formed in the melt by subsequent crystallization [7,10,11,12].

When diblock copolymers are composed of two crystallizable blocks, the final morphology still depends on the competition between phase separation and crystallization, and if the two blocks crystallize at different temperatures, the crystallization of the first block may define the final morphology, or be modified by the subsequent crystallization of the other block [3,4,5,6,7,13,14,15,16,17,18,19]. Confined crystallization may occur even when the two blocks are miscible in the melt because one block may crystallize within the lamellar crystals of the other block previously formed, giving lower crystallization and melting temperatures [1,3,4,6,7,17,18,19].

The crystallization behavior of crystalline BCPs containing poly(ethylene oxide) (PEO), poly(ε-caprolactone) (PCL), polyethylene (PE), poly(L-lactide) (PLLA) and hydrogenated polynorbornene (hPN) have been extensively investigated [4,13,14,15,16,20,21,22,23,24,25,26]. In contrast, BCPs containing blocks based on crystallizable stereoregular polyolefin have received less attention because of the difficulty of the synthesis and the intrinsic limitations of the living polymerization methods available to date to ensure high stereochemical control in living olefin polymerization. Therefore, the morphologies that develop in crystalline BCPs composed of crystallizable polyolefins blocks, and the relationships between crystallization and phase separation in the melt, have been mainly studied in BCPs containing crystallizable polyethylene (PE) blocks synthesized by hydrogenation of BCPs containing 1,4-polybutadiene blocks prepared by classic anionic living polymerization [3,7,10,11,12,27,28,29,30,31,32,33,34,35,36,37,38,39,40,41,42]. This has resulted in highly defective PE blocks with low melting temperature (about 90 °C) containing high amounts of constitutional defects of 1-butene units arising from hydrogenation of 1,2-butadiene units present as defects in the precursor 1,4-polybutadiene blocks [27,28,29,30,31,32,33,34,35,36,37,38,39,40,41,42].

More recently, BCPs containing blocks based on crystallizable stereoregular polyolefins, in particular isotactic propylene (iPP) and syndiotactic polypropylene (sPP), and linear PE, have been successfully synthesized due to the development of organometallic catalysts able to promote stereoselective and living olefin polymerization [43,44,45,46,47]. This has allowed the synthesis and the study of the crystallization and phase separation of BCPs containing crystallizable isotactic or syndiotactic polypropylene linked to amorphous blocks or to crystalline linear PE [17,18,19,48,49,50,51,52,53].

Block copolymers containing crystallizable blocks have aroused great interest as thermoplastic elastomers due to the improved mechanical properties and thermal stability with respect to conventional elastomers. The possibility of synthesizing BCPs containing crystallizable iPP or sPP blocks linked to amorphous rubbery blocks or crystallizable PE block is of great interest for producing novel rubbery materials with remarkable mechanical strength, in which the polymorphic behaviors of iPP or sPP and their copolymers [54,55,56], and the crystal morphology and phase transformations occurring during deformation, play a role [57,58,59,60,61,62,63,64,65,66].

In this paper, we report a study of the structure and morphology of samples of crystalline–crystalline BCP composed of blocks of crystallizable polyethylene (PE) and syndiotactic polypropylene (sPP) (PE-b-sPP) having different block lengths. We show that the final morphology that develops upon crystallization from the melt is characterized by strict alternation of amorphous and crystalline layers, the latter being composed of two different crystalline phases (sPP and PE) having different periodicities, which could be controlled by the relative molecular mass of the two blocks.

## 2. Materials and Methods

Samples of PE-b-sPP were prepared with a living organometallic catalyst, bis[N-(3-tert-butylsalicylidene)-2,3,4,5,6-pentafluoroanilinato]-titanium(IV) dichloride (from MCAT, Donaueschingen, Germany), activated with methylalumoxane (MAO) (from Lanxess, Cologne, Germany) [43,50]. The molecular mass and the polydispersity of the sample were determined by gel permeation chromatography (GPC), using a Polymer Laboratories GPC220 apparatus equipped with a Viscotek 220R viscometer (Agilent Company, Santa Clara, CA, USA), on polymer solutions in 1,2,4-trichlorobenzene at 135 °C. The molecular structure was analyzed by ^13^C NMR spectroscopy using a Varian VXR 200 spectrometer (Varian Company, Palo Alto, CA, USA).

The molecular characteristics of the samples of PE-b-sPP are reported in Table 1. The sample PE-*b*-sPP-1 has PE and sPP blocks with similar molecular masses (*M*_n(sPP)_ = 10200 and *M*_n(PE)_ = 9800), with a 53% volume fraction of the sPP block (*f*_sPP_), whereas the sample PE-*b*-sPP-2 has a higher total molecular mass (*M*_n_ = 64000), and an sPP block longer than the PE block (*M*_n(sPP)_ = 46700 and *M*_n(PE)_ = 17300) with a volume fraction of sPP of *f*_sPP_ = 75%. Both samples are characterized by a narrow polydispersity with *M*_w_/*M*_n_ = 1.2. The molecular masses of the blocks were estimated from the total *M*_n_ and the weight% (*wt*%) of PE or sPP, determined by ^13^C NMR, such that *M*_n(PE)_ = *M*_n_ × *w*_PE_ and *M*_n(sPP)_ = *M*_n_ − *M*_n(PE)_. The volume fractions of the blocks were calculated from the molecular masses of the two blocks *M*_n(PE)_ and *M*_n(sPP)_ and the densities of crystalline PE (0.997 g/cm^3^) and sPP (0.90 g/cm^3^) [67], such that *f*_PE_ = (*M*_n(PE)_/0.997)/(*M*_n(sPP)_/0.90 + *M*_n(PE)_/0.997).

Calorimetric measurements (DSC-822, Mettler Toledo, Columbus, OH, USA) were performed under flowing N_2_ at heating and cooling rates of 10 °C/min. 

X-ray powder diffraction profiles were obtained with Ni-filtered CuKα radiation with an Empyrean diffractometer (Malvern Panalytical, Worcestershire, UK). Diffraction profiles were also recorded in situ at different temperatures during heating and cooling from the melt at approximately 10 °C/min using an attached TTK non-ambient stage (Anton Paar KG, Graz, Austria). The sample was heated from 25 °C up to the melt at 150 °C at nearly 10 °C/min and the diffraction profiles were recorded every 5 degrees starting from 105 °C up to 150 °C. Then, the sample was cooled from the melt at 150 °C down to 25 °C, still at 10 °C/min, and the diffraction profiles were recorded every 5 degrees during cooling. The temperature was kept constant while recording of each diffraction profile during both heating and cooling.

Small-angle X-ray scattering (SAXS) profiles were collected using an evacuated high performance SAXS instrument “SAXSess” (Anton Paar KG, Graz, Austria), which is a modification of the so-called “Kratky compact camera” [68,69]. Data collection was performed in the slit collimation configuration with the SAXSess camera attached to a conventional X-ray source (CuKα, wavelength λ = 1.5418 Å). The scattered radiation was recorded on a BAS-MS imaging plate (Fujifilm) in a configuration which allowed simultaneous recording of WAXS and SAXS data and was processed with a digital imaging reader (Cyclone Plus Phosphor Imager, Perkin-Elmer, Waltham, MA, USA) at a resolution in the small angle region of 2π/*q*_min_ ≈ 60 nm, where *q* = 4πsinθ/λ is the scattering vector and 2θ the scattering angle, with *q*_min_ = 0.1 nm^−1^ being the minimum accessible value of the scattering vector permitted by our collimation setup. After subtraction for dark current, the empty sample holder, and a constant background due to thermal density fluctuations, the slit-smeared data in the SAXS region (for *q* < 4 nm^−1^) were de-convolved with the primary beam intensity distribution using the software SAXSquant 2.0 (Anton Paar KG, Graz, Austria) to obtain the corresponding pinhole scattering (desmeared) intensity distribution. The SAXS profiles were recorded at different temperatures starting from the melt and cooling down to room temperature. The desmeared SAXS intensities were then used to calculate the normalized self-correlation function of electron density fluctuations γ(*r*).

Thin films (thickness lower than 50 nm) of the samples of PE-b-sPP were prepared for transmission electron microscope (TEM) observation by casting at room temperature on microscope glass slides from a *p*-xylene solution (0.2 wt%–0.5 wt%). The so-obtained thin films were carbon-coated under vacuum in an EMITECH K950X evaporator (Quorum Technologies Ltd., Lewes, UK). To improve contrast, the thin films were decorated with gold nanoparticles by vacuum evaporation and condensation. After evaporation, gold condensates and deposits formed mainly at the amorphous-crystalline interface of the semicrystalline lamellae allowing better visualization of the crystalline phases. The films were then floated off on water with the help of a poly(acrylic acid) backing and mounted on copper grids.

Transmission electron microscope (TEM) images in bright-field mode were taken in an FEI TECNAI G^2^ 200 kV S-TWIN microscope equipped with 4K camera (electron source with LaB_6_ emitter) (FEI Company, Dawson Creek Drive, Hillsboro, OR, USA). Bright-field (BF) TEM images were acquired at 120 kV using a spot size equal to 3, integration time 1 s. 

## 3. Results and Discussion

**Figure 1:**
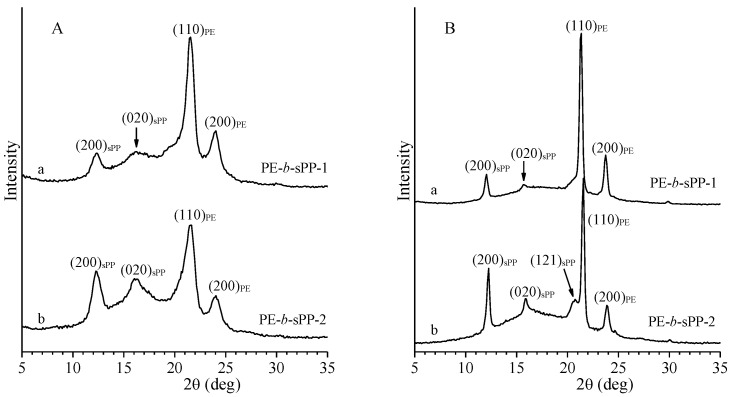
X-ray powder diffraction profiles of as-prepared samples (**A**) and of samples crystallized from the melt by compression molding and cooling down to room temperature at 10 °C/min (**B**) of the BCP samples PE-b-sPP-1 (a) with *f*_sPP_ = 53% and PE-b-sPP-2 (b) with *f*_sPP_ = 75%. The (200)_sPP_, (020)_sPP_ and (121)_sPP_ reflections of form I of sPP at 2θ = 12.2°, 16°, and 21°, and the (110)_PE_ and (200)_PE_ reflections at 2θ = 21.5° and 24° of the orthorhombic form of PE are indicated.

The X-ray powder diffraction profiles of the as-prepared (as-precipitated from the polymerization solution) samples of PE-b-sPP block copolymers of Table 1 are reported in Figure 1A. The presence of the 200 and 020 reflections of form I of sPP at 2θ = 12° and 16° and of the 110 and 200 reflections at 2θ = 21.5° and 24° of PE, indicates that in both samples sPP crystallizes in the most stable helical form I [70,71] and PE crystallizes in the stable orthorhombic form [72]. 

The intensities of the reflections of sPP crystals in the diffraction profile of the sample PE-b-sPP-2 (profile b of Figure 1A) were higher than those in the profile of the sample PE-b-sPP-1, according to the higher volume fraction of the sPP block in the sample PE-b-sPP-2 (Table 1). The diffraction profiles of Figure 1A also indicated that the degree of crystallinity of the as-prepared sample PE-b-sPP-1, having lower molecular mass (49%), is slightly higher than that of the sample PE-b-sPP-2 (40%).

The X-ray diffraction profiles of samples crystallized from the melt by compression molding and cooling down to room temperature at 10 °C/min are shown in Figure 1B. The diffraction profiles of both samples still present the 200 and 020 reflections of form I of sPP at 2θ = 12° and 16°, and the 110 and 200 reflections at 2θ = 21.5° and 24° of the orthorhombic form of PE. The reflections are sharper than those in the diffraction profiles of the as-prepared samples of Figure 1A, making visible also the 121 reflection of form I of sPP at 2θ = 21° (profile b of Figure 1B). The diffraction profiles of Figure 1B also indicate that the melt-crystallized samples show slightly higher crystallinity.

**Figure 2:**
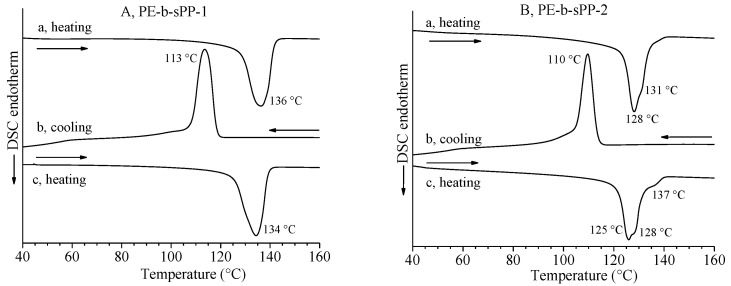
DSC curves recorded during heating of the as-prepared samples (a), cooling from the melt (b) and successive second heating of the melt-crystallized samples (c) of samples PE-b-sPP-1 (**A**) with *f*_sPP_ = 53% and PE-b-sPP-2 (**B**) with *f*_sPP_ = 75%. All thermograms have been recorded at a scanning rate of 10 °C/min.

The DSC curves of the two samples of PE-b-sPP are reported in Figure 2. The thermograms recorded during the first heating of the as-prepared samples, and the second heating of the samples crystallized from the melt by cooling at 10 °C/min (curves a and c of Figure 2), show for both samples only one broad endothermic peak with shoulders at high temperatures, indicating melting of PE and sPP crystals in the same range of temperature. In the cooling curves b of Figure 2, only one exothermic peak, corresponding to the almost simultaneous crystallization of PE and sPP, is observed for both samples. The symmetric sample PE-b-sPP-1, with similar lengths of PE and sPP blocks, shows a melting temperature (134–136 °C, curves a,c of Figure 2A) and crystallization temperature (113 °C, curve b of Figure 2A) higher than those of the asymmetric sample PE-b-sPP-2 with a longer sPP block (melting peak at 126–128 °C, curves a,c of Figure 2B and crystallization peak at 110 °C, curve b of Figure 2B). 

**Figure 3:**
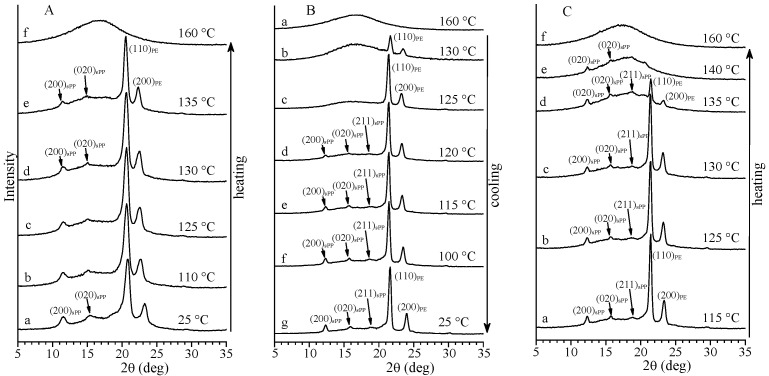
X-ray powder diffraction profiles of the sample PE-*b*-sPP-1 with *f*_sPP_ = 53% recorded at different temperatures during first heating (a–f) of the as-prepared sample up to the melt (**A**), during cooling (a–g) from the melt down to room temperature (**B**), and during successive heating (a–f) of the melt-crystallized sample up to the melt (**C**). The (200)_sPP_, (020)_sPP_ and (211)_sPP_ reflections of form I of sPP at 2θ = 12°, 16° and 18.8°, respectively, and the (110)_PE_ and (200)_PE_ reflections of the orthorhombic form of PE at 2θ = 21° and 24°, respectively, are indicated.

**Figure 4:**
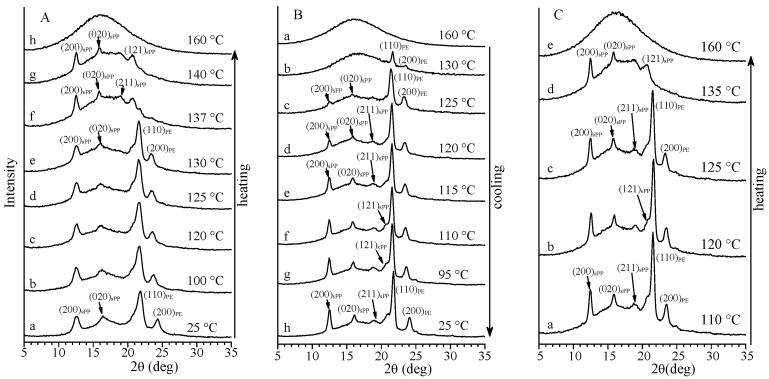
X-ray powder diffraction profiles of the sample PE-*b*-sPP-2 with *f*_sPP_ = 75% recorded at different temperatures during first heating (a–h) of the as-prepared sample up to the melt (**A**), during cooling (a–h) from the melt down to room temperature (**B**), and during successive heating (a–e) of the melt-crystallized sample up to the melt (**C**). The (200)_sPP_, (020)_sPP_, (211)_sPP_ and (121)_sPP_ reflections of form I of sPP at 2θ = 12°, 16°, 18.8° and 20.7°, respectively, and the (110)_PE_ and (200)_PE_ reflections of the orthorhombic form of PE at 2θ = 21° and 24°, respectively, are indicated.

The X-ray diffraction profiles of samples of PE-b-sPP-1 and PE-b-sPP-2, recorded at different temperatures during heating and cooling from the melt down to room temperature, are reported in Figure 3 and Figure 4, respectively. In the as-prepared specimen of the symmetric sample PE-b-sPP-1 with *f*_sPP_ = 53% (Figure 3A), the PE and sPP blocks seem to melt almost simultaneously as the intensities of the 110 and 200 reflections of PE at 2θ = 21° and 24°, and of the 200 and 020 reflections of sPP at 2θ = 12° and 16°, do not decrease up to 135 °C and disappear almost at the same temperature. This agrees with the single endothermic peak present in the DSC heating curve a of Figure 2A. In the melt-crystallized specimen of the sample PE-b-sPP-1 (Figure 3C), instead, the intensities of the 110 and 200 reflections of PE decrease at temperatures higher than nearly 130 °C, while the intensities of the 200 and 020 reflections at 2θ = 12 and 16° of sPP do not change up to nearly 135 °C. This indicates that during heating of the melt-crystallized sample PE-b-sPP-1, the PE block melts before the sPP block. This is more evident in the sample PE-b-sPP-2 having the longer sPP block (*f*_sPP_ = 75%) of Figure 4A,C. In both as-prepared (Figure 4A) and melt-crystallized (Figure 4C) specimens of the sample PE-b-sPP-2, the intensities of the 110 and 200 reflections of PE at 2θ = 21° and 24° decrease at temperatures higher than 130 °C, and disappear at 135–137 °C in the diffraction profiles recorded during heating (Figure 4A,C), while the 200 and 020 reflections at 2θ = 12° and 16° of sPP are still present (profiles f of Figure 4A and d of Figure 4C). This indicates that even though the DSC heating curves of the as-prepared and melt-crystallized samples of PE-b-sPP-2 present single broad endotherms (curves a and c of Figure 2B), crystals of PE melt before those of sPP, and, therefore, in the asymmetric melting peaks of the sample PE-b-sPP-2 of Figure 2B (curves a and c), the peaks at low temperature of 125–128 °C correspond to the melting of the PE block and the shoulders at 131–137 °C correspond to the melting of the sPP block (curves a, c of Figure 2B). 

It is worth noting that during heating of the as-prepared specimen of the sample PE-b-sPP-2, a new reflection at 2θ = 18.8° appears at 137 °C (profile f of Figure 4A). This reflection corresponds to the 211 reflection typical of the ordered form I of sPP [70,71,73], which is absent in the diffraction profiles of the as-prepared samples at room temperature (Figure 1 and profiles a of Figure 3A and Figure 4A). This indicates that the as-prepared samples are crystallized in disordered modifications of form I [70,73], characterized by disorder in the alternation of right-handed and left-handed 2/1 helical chains of sPP along the *a* and *b* axes of the orthorhombic unit cell of form I [70,73]. Annealing at high temperatures that occurred during heating of the as-prepared sample and recording the diffraction profiles, allows development of more ordered modifications of form I of sPP, characterized by a higher order in the alternation of enantiomorphous helices along both axes of the unit cell [70,71,73].

The diffraction profiles recorded during cooling from the melt at 160 °C down to room temperature of Figure 3B and Figure 4B indicate that, starting from the amorphous halo, the 110 and 200 reflections of PE at 2θ = 21° and 24° appear first, already at 125–120 °C (profiles b of Figure 3B and Figure 4B), before the 200 and 020 of sPP at 2θ = 12° and 16° that appear only at lower temperatures (profile d of Figure 3B and profiles c–d of Figure 4B). Therefore, even though the DSC cooling scans show only a single crystallization peak (curves b of Figure 2), during the slow cooling and the isothermal necessary to record the diffraction profiles, the PE block crystallizes first at high temperatures (nearly 120–125 °C). The intensities of the reflections of both sPP and PE increase and became sharper on further cooling. Moreover, the diffraction profiles of Figure 3B and Figure 4B also show that, upon cooling from the melt besides the 200 and 020 reflections of sPP, the 211 reflection at 2θ = 18.8° of the ordered form I of sPP also develops [70,71,73] (profiles f, g of Figure 3B and d–h of Figure 4B). As discussed above, this reflection is absent in the diffraction profiles of the as-prepared samples or the compression molded samples of Figure 1. This indicates that the as-prepared and melt-crystallized compression molded samples are crystallized in disordered modifications of form I [70,73]. The slow crystallization from the melt of Figure 3B and Figure 4B induces, instead, the crystallization of a more ordered modification of form I of sPP, characterized by a higher order in the perfect alternation of enantiomorphous helices along both axes of the unit cell [70,71,73].

**Figure 5:**
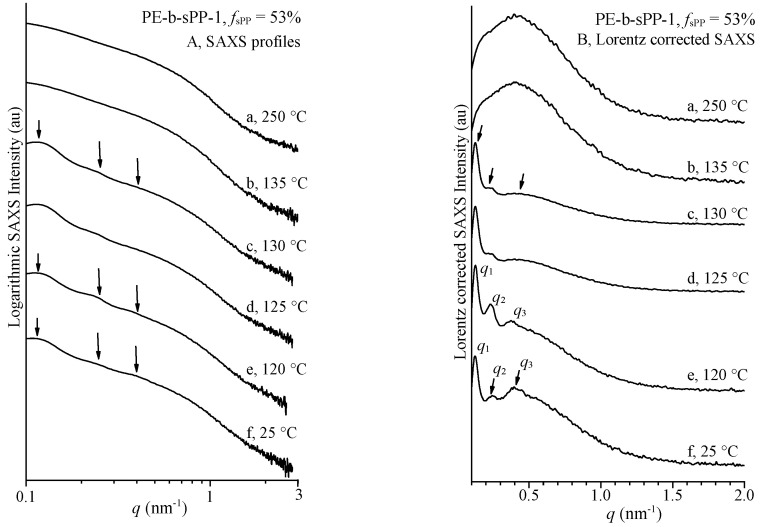
SAXS profiles (**A**) and Lorentz-corrected profiles (**B**) of the sample PE-*b*-sPP-1 with *f*_sPP_ = 53% recorded at different temperatures (a–f) during cooling from the melt down to room temperature. The three correlation peaks *q*_1_, *q*_2_ and *q*_3_, corresponding to lamellar stacks of PE and sPP, are indicated.

SAXS experiments were performed on the symmetric sample PE-b-sPP-1 with *f*_sPP_ = 53%, and high and comparable crystallinities of the two blocks, to provide information about the morphology that develop by cooling from the melt and after the crystallization of the PE and sPP blocks. The SAXS profiles of the sample PE-b-sPP-1 recorded at different temperatures, starting from the melt at 250 °C and cooling down to room temperature, are reported in Figure 5A. The Lorentz-corrected SAXS profiles are reported in Figure 5B. The SAXS profile of the melt recorded at 250 °C (profiles a of Figure 5) is essentially featureless and not informative. This does not necessarily indicate that the melt is homogeneous, but is due to the very small electron density difference between the PE and sPP blocks in the melt that prevents observation of the eventual phase-separated structure.

The possible phase separation in the melt for PE-b-sPP BCPs has been discussed in [50]. According to mean-field theory, the order-disorder transition for symmetric BCPs occurs at a fixed interaction strength for calculated values of χ*N* = 10.5, where χ is the Flory–Huggins interaction parameter, and *N* is the total number of equivalent segments that constitute the macromolecules of the blocks of the BCP [50]. For non-symmetric BCPs, the phase separation transition occurs for higher values of χ*N*. For polyolefin-based BCPs, the equivalent segments are assumed as a portion of chains having the density of four CH_2_ units (four carbon atoms segment). The Flory–Huggins interaction parameter χ between sPP and PE has been determined in [50] as: χ = 6.2/*T* − 0.0053, with *T* the absolute temperature. For the sample PE-b-sPP with total *M*_n_ = 20000 and *f*_sPP_ = 53%, the total number of equivalent segments *N* that constitute the macromolecules of the blocks is *N* = *M*_n_/56 = 357 (where 56 is the molecular mass of the four CH_2_ carbon atoms segment). Therefore, for this sample, the order-disorder transition temperature *T*_ODT_ may be calculated from χ*N* ≥ 10.5 = (6.2/*T* − 0.0053)357, and is expected to be lower than 0 °C. This indicates that the crystallization of the sample PE-b-sPP-1 most likely takes place from a homogeneous melt. 

**Figure 6:**
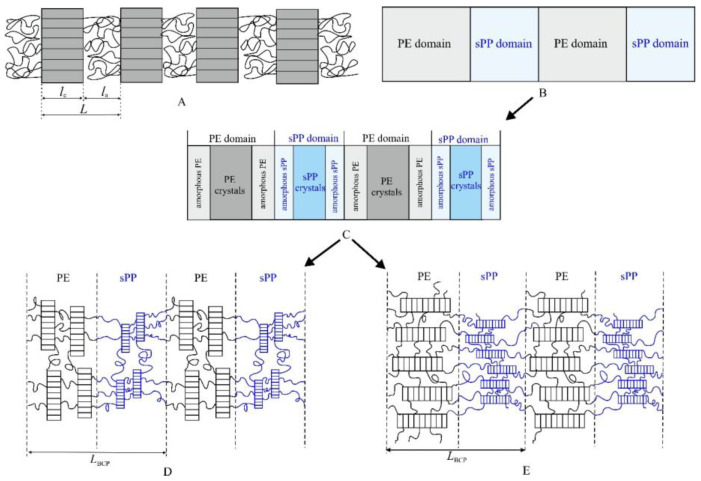
Schematic models of morphology that may develop in the sample PE-b-sPP-1 with *f*_sPP_ = 53%, based on SAXS profiles of Figure 5. (**A**) Stacks of crystalline lamellae of PE and/or sPP alternating with amorphous chains; (**B**) Possible lamellar morphology of the nearly symmetric BCP in the melt with alternating amorphous lamellar domains of PE and sPP; (**C**) Possible morphology that develops upon cooling from the melt, characterized by alternating sPP and PE domains, which include stacks of crystalline lamellae of sPP and PE, respectively, separated by the corresponding amorphous regions of sPP and PE; (**D**,**E**) Details of the morphology in C showing two possible limit orientations of the chain axes in the crystalline domains, normal (**D**) and parallel (**E**) to the lamellar domain interface (**D**).

Starting from the featureless SAXS profile of the melt at 250 °C, upon cooling, three correlation peaks at values of the scattering vector *q*_1_ = 0.12 nm^−1^, *q*_2_ = 0.24 nm^−1^ and *q*_3_ = 0.4 nm^−1^ appear in the SAXS profiles of Figure 5 already at 130 °C. Correspondingly, the wide-angle diffraction profiles of Figure 3B show that crystallization of PE and sPP occur by cooling at temperatures below 130 °C. With further decrease in temperature, the three correlation peaks became more intense and appear well-resolved at room temperature where crystals of PE and sPP are well-formed. The three correlation peaks correspond to the sum of scattering from the sPP and PE semicrystalline blocks and indicate development of a lamellar morphology, where the lamellar crystals of PE and/or sPP alternate with amorphous layers, as shown schematically in Figure 6A. 

The formation of lamellar morphology in the sample PE-b-sPP-1, characterized by a similar length of the two blocks, is compatible with both the hypotheses of crystallization from a homogeneous melt and from a segregated melt (Figure 6B). In a symmetric block copolymer a melt lamellar morphology, where sPP layers alternate with PE layers, would be expected (Figure 6B) [9]. In this case crystallization would occur confined to the preformed lamellar domains of sPP and PE, giving a global lamellar morphology, where stacks of crystalline lamellae of PE and sPP grow within the corresponding lamellar domains of the two blocks (Figure 6C). On the other hand, if phase separation is driven by crystallization, a morphology characterized by the alternation of sPP and PE domains, which include stacks of crystalline lamellae of sPP and PE, respectively, separated by the corresponding amorphous regions of sPP and PE, is expected (Figure 6C). In both cases, consecutive crystalline layers of sPP and PE are separated by amorphous layers of chains of the two blocks and the crystalline lamellae would assume different orientations within the lamellar domains of the two blocks (Figure 6C). 

This equilibrium morphology, that involves strict alternation of amorphous and crystalline layers, has been described in the case of crystallization of one block in crystalline-amorphous diblock copolymers [1,7,10,31,32,33,74]. In the case of crystalline-crystalline PE-b-sPP block copolymers, the alternating crystalline domains separated by amorphous layers are made of two different crystalline phases (sPP and PE lamellae) (Figure 6C), characterized, probably, by different periodicities, with each crystalline layer being sandwiched by its own amorphous phase (sPP or PE) (Figure 6C). According to models proposed for amorphous-crystalline diblock copolymers, two limit orientations of the chain axes in the crystalline domains are possible. The chain axis may be normal to the lamellar interface, as in the model of Figure 6D, or parallel to the lamellar interface as in the model of Figure 6E. Generally, the different directions of the chain axes in lamellar systems of crystalline BCPs have been attributed to different states of the system and crystallization conditions, such as crystallization from a homogeneous melt or weakly segregated melt, that should favor orientation of the chain axes perpendicular to the lamellar interface [7,31,33], or crystallization from a segregated melt that should favor orientation of the chain axes parallel to the lamellar interface [1,7,28,29,75]. In any case, the two models of Figure 6D,E correspond to two limit orientations of the chain axes and of folding directions, because, in the absence of external bias that induces fiber or single crystal orientation of the crystalline phases [17,18,19,38,48,49], the crystalline lamellae assume different, almost random, orientations within the lamellar domains of the two blocks.

Details of the morphology with determination of the lamellar parameters, that is, the thicknesses of the two crystalline lamellae of PE and sPP and of the amorphous layers and of the values of the periodicity, and assignment of the observed periodicities of PE or sPP lamellar stacks, can be obtained by analyzing the WAXS and SAXS profiles recorded during cooling. In the SAXS profiles of Figure 5, the three correlation peaks at *q*_1_ = 0.12 nm^−1^, *q*_2_ = 0.24 nm^−1^ and *q*_3_ = 0.4 nm^−1^, appear already at 130 °C, during cooling from the melt, when only the PE crystals are probably formed, as indicated by the WAXS profiles of Figure 3B that show starting of crystallization of sPP only at 120 °C. This probably indicates that the SAXS correlation peaks can be attributed mainly to the formation of stacks of crystalline lamellae of PE. Therefore, the first correlation peak at *q*_1_ = 0.12 nm^−1^ may be interpreted as the first-order diffraction of a monodimensional lattice of periodicity (the long period) calculated by direct application of the Bragg law *L*_B_ = 2π/*q*_1_ = 52 nm. This monodimensional lattice corresponds to stacks of parallel crystalline lamellae of PE, spaced by *L*_B_ alternating with amorphous layers, as in Figure 6A. The second correlation peak occurs at *q*_2_ ≈ 2*q*_1_ = 0.24 nm^−1^ and may correspond to the second-order diffraction of the monodimensional lattice, confirming the formation of almost ideal stacks of parallel crystalline lamellae of PE of long period *L*_B_. The third correlation peak at *q*_3_ = 0.4 nm^−1^ ≈ 3*q*_1_ may correspond to the third-order diffraction of the PE lamellar stacks or may be due to the formation of crystalline lamellae of sPP with lower periodicity *L*_B_ = 2π/*q*_3_ = 16 nm. The latter hypothesis is based on the observation that, in the SAXS profiles of Figure 5B, the correlation peak at *q*_3_ is better defined at low temperatures when crystallization of sPP proceeds. In any case, at temperatures lower than 120 °C, the contribution of sPP crystals is not negligible. Therefore, at 130 °C, the morphology may be described by the simple model of Figure 6A, where crystalline layers of PE (constituted by stacks of crystalline lamellae) alternate with amorphous layers, which are composed of the amorphous phase of PE and the amorphous (melt) sPP. At lower temperature, crystallization of sPP proceeds and stacks of crystalline lamellae of sPP grow within the sPP domain (Figure 6C).

The values of the scattering vector *q* of the correlation peaks observed in the SAXS profiles of Figure 5B at different temperatures during cooling, and the values of the long period *L*_B_ calculated from the first correlation peak *L*_B_ = 2π/*q*_1_, are reported in Table 2. Assuming the model of PE lamellar stacks of Figure 6A, from the values of the long period *L*_B_, evaluated from the first correlation peak *q*_1_ and the degree of crystallinity, evaluated from the WAXS diffraction profiles of Figure 3B, the average value of the thickness of the crystalline lamellae *l*_c_ of PE has been calculated as *l*_c_ * = φ_c_(WAXS) × *L*_B_, where φ_c_(WAXS) is the volume fraction of the crystalline phase [1,76], approximately evaluated from the degree of crystallinity extracted from the WAXS profiles *x*_c_ and the density of the crystalline and amorphous phases as: φ_c_ = *x*_c_ *d*_c_^−1^[*x*_c_ *d*_c_^−1^ + (1 − *x*_c_) *d*_a_^−1^], where *d*_c_ = 0.997 g/cm^3^ and *d*_a_ = 0.850 g/cm^3^ are the densities of crystals of PE in the orthorhombic form and of the amorphous PE at 25 °C, respectively [67]. The average values of the thickness of the PE amorphous layers between PE lamellae has been calculated as *l*_a_ *= *L*_B_ − *l*_c_ *. The values of the thicknesses of the crystalline and amorphous layers *l*_c_ * and *l*_a_ * in the PE domains are reported in Table 2.

To gain further details of the bulk morphology that develops in the sample of PE-b-sPP BCP upon crystallization of the two blocks revealing the different periodicities of the stacks of crystalline lamellae of PE and sPP included in the PE and sPP lamellar domains of Figure 6C, the self-correlation function of electron density fluctuations has been extracted from the SAXS data of Figure 5. The normalized self-correlation function of electron density fluctuations γ(*r*) has been calculated from the observed SAXS intensity *I*_obs_(*q*) by the following equation relative to an ideal two phases lamellar morphology [76]:

$$\gamma \left(r\right)=\frac{\int_{0}^{\infty }q^{2}I_{c}\left(q\right)\cos\left(qr\right)dq}{\int_{0}^{\infty }q^{2}I_{c}\left(q\right)dq}$$

where *r* is the correlation distance along the monodimensional lattice of Figure 6A, and *I*_c_(*q*) is the observed desmeared SAXS intensity after subtraction for a residual background *I*_back_:

*I*_c_(*q*) = *I*_obs_(*q*) − *I*_back_

**Figure 7:**
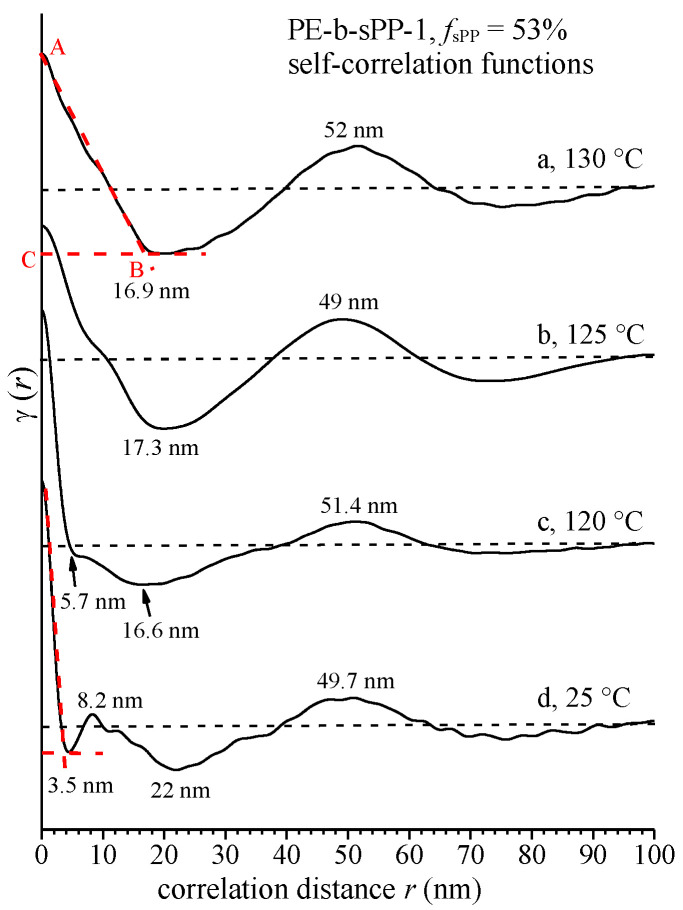
Normalized self-correlation functions of electron density fluctuations γ(*r*) calculated from the SAXS intensities of the sample PE-b-sPP-1 with *f*_sPP_ = 53% of Figure 5, recorded at different temperatures (a–d) during cooling from the melt. The main correlation triangle ABC, and the average values of periodicity *L*, and of thicknesses of crystalline or amorphous layers of the layer structure of Figure 6C and Figure 8, are indicated.

For high values of the scattering vector (*q* > 1.5 nm^−1^), this SAXS intensity has been fitted to the Porod law *I_c_*(*q*) = *K_p_ q*^−4^, assuming that the interphase surface between the two phases, the crystalline lamellae and the amorphous layers, is sharp [76,77,78]. The normalized self-correlation functions of the electron density fluctuations γ(*r*), calculated from the SAXS intensities of the sample PE-b-sPP-1 of Figure 5, recorded at different temperatures during cooling from the melt, are reported in Figure 7. Only the data at temperatures at which crystallization had already begun are shown.

The correlation functions present the typical shape expected for a lamellar morphology of Figure 6A [1]. In fact, in the simple cases, at temperatures of 130 °C and 125 °C (curves a and b of Figure 7), the correlation functions present a main maximum at a correlation distance around 50 nm, corresponding to the long period *L*, and a main minimum at correlation distance around 17 nm, corresponding to the minimum thickness of layers in the stacks (thickness of amorphous *l*_a_ or crystalline *l*_c_ layers) (curves a, b of Figure 7). More precisely, the lamellar parameters have been extracted from the “self-correlation triangle” ABC of the correlation functions (Figure 7), considering that the minimum layer thickness in the stacks corresponds to the abscissa of the point B of the correlation triangle ABC (curve a of Figure 7), and corresponds to the thickness of the amorphous layer *l*_a_, if the degree of crystallinity evaluated from the WAXS profiles is higher than 50%, or to the thickness of the crystalline lamellae *l*_c_, if the crystallinity is lower than 50%. Then, the larger thickness, *l*_c_ or *l*_a_, is calculated as *l*_c_ (*l*_a_) = *L* − *l*_a_ (*l*_c_). The values of the long period *L*_CF,_ and the thicknesses of crystalline *l*_c(CF)_ and amorphous *l*_a(CF)_ layers determined from the correlation functions of Figure 7, are reported in Table 2, and compared with the values obtained from the SAXS correlation peaks by direct application of the Bragg law (*L*_B_, *l*_c_ * and *l*_a_ *). The values obtained with the Bragg law and the correlation functions at 130 and 125 °C are very similar. 

**Figure 8:**
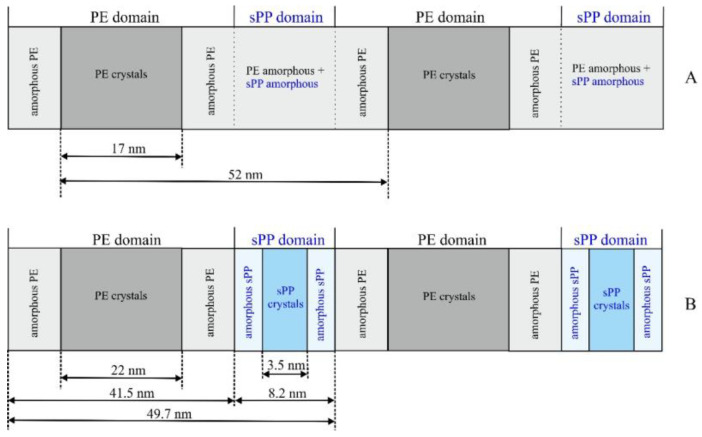
Models of the morphology of the sample PE-b-sPP-1 with *f*_sPP_ = 53% that develops by crystallization upon cooling from the melt, at 125–130 °C when mainly PE crystals contribute (**A**), and at room temperature when both PE and sPP crystals are well-formed (**B**). The values of the thickness of the crystalline lamellae of PE and sPP and of the amorphous layers and of the periodicities, are indicated.

As discussed above, at temperatures higher than 120 °C, when only PE crystals are formed (as indicated by the WAXS profiles of Figure 3B), and the contribution of sPP crystals is negligible, the SAXS intensity is essentially due to the fluctuation of the electron density for the formation of crystals of PE and the values of the long period and the thicknesses of the amorphous and crystalline layers, evaluated by both the Bragg law and correlation functions (profiles c, d of Figure 5 and curves a, b of Figure 7), refer essentially to stacks of crystalline lamellae of PE alternating with the amorphous layer, as in Figure 6A. Therefore, since the degrees of crystallinity at 130 and 125 °C are lower than 50%, the lowest thickness of nearly 17 nm (Table 2 and Figure 7a,b) corresponds to the thickness of the crystalline lamellae of PE, and the larger thickness of 33–34 nm, corresponds to the thickness of the amorphous phase that includes the amorphous phase of PE and the amorphous sPP still in the melt state. A schematic model of the morphology that developed at 125–130 °C, when mainly PE crystals contribute, with assignment of the observed periodicities, is shown in Figure 8A. The long period of 49–52 nm (Table 2) corresponds to the average distance between the crystalline lamellae of PE. 

At lower temperatures, when crystallization of sPP proceeds, the contribution of sPP is no longer negligible, and the self-correlation functions assume a more complex shape (curves c, d of Figure 7), showing new minimum and maximum, and deviations from the shape of the ideal two-phase lamellar morphology of Figure 6A and Figure 8A. At room temperature, the correlation function presented a new minimum at a correlation distance of 3.5 nm, and a new maximum at a distance of 8.2 nm (curve d of Figure 7), that are probably due to the formation of lamellar crystals of sPP. A model of the morphology of the BCP that develops at room temperature, when both PE and sPP crystals are well formed, is reported in Figure 8B. The minimum thickness of the lamellar morphology is, this time, 3.5 nm, that can be interpreted as the thickness of the crystalline lamellae of sPP (*l*_c(sPP)_). The distance of 8.2 nm may be interpreted as the thickness of the whole sPP domains *L*_sPP_ containing amorphous and crystalline layers of sPP (Figure 8B). The second minimum, at a correlation distance of 22 nm at room temperature (curve d of Figure 7), corresponds to the thickness of the crystalline lamellae of PE (Figure 8B), according to the value of nearly 17 nm achieved at 120–130 °C (Figure 8A). The maximum in the correlation curve at room temperature at a correlation distance of 49.7 nm (curve d of Figure 7), which has been interpreted as the long period of the PE lamellar stacks at 120–130 °C (curves a, b of Figure 7), may be interpreted as the distance between the crystalline PE lamellae, which corresponds to the periodicity of the lamellar BCP *L*_BCP_ = *L*_PE_ + *L*_sPP_ = 49.7 nm, where *L*_PE_ and *L*_sPP_ are the thicknesses of the PE and sPP lamellar domains, each domain being composed of crystalline and amorphous layers *L*_PE_ = *l*_c(PE)_ + 2*l*_am(PE)_ and *L*_sPP_ = *l*_c(sPP)_ + 2*l*_am(sPP)_ (Figure 8B). 

The values of the thicknesses of the PE and sPP lamellar domains, *L*_PE_ and *L*_sPP_, and of the thicknesses of the crystalline and amorphous layers in the PE and sPP domains in the model of Figure 8B, *l*_c(PE)_, *l*_am(PE)_, *l*_c(sPP)_ and *l*_am(sPP)_, are reported in Table 2. The thicknesses of the amorphous PE and sPP layers were calculated from the values of thickness of the corresponding domains and of the global periodicity *L*_BCP_ (Table 2 and Figure 8). In detail, 2*l*_am(sPP)_ = *L*_sPP_ − *l*_c(sPP)_ = 8.2 − 3.5 = 4.7 nm, and, since *L*_BCP_ = *L*_PE_ + *L*_sPP_ = (*l*_c(PE)_ + 2*l*_am(PE)_) + (*l*_c(sPP)_ + 2*l*_am(sPP)_) = 49.7 nm, 2*l*_am(PE)_ = *L*_BCP_ − *l*_c(PE)_ − (*l*_c(sPP)_ + 2*l*_am(sPP)_) = 49.7 − 22 − 8.2 = 19.5 nm and *L*_PE_ = 22 + 19.5 = 41.5 nm (Table 2 and Figure 8B).

**Figure 9:**
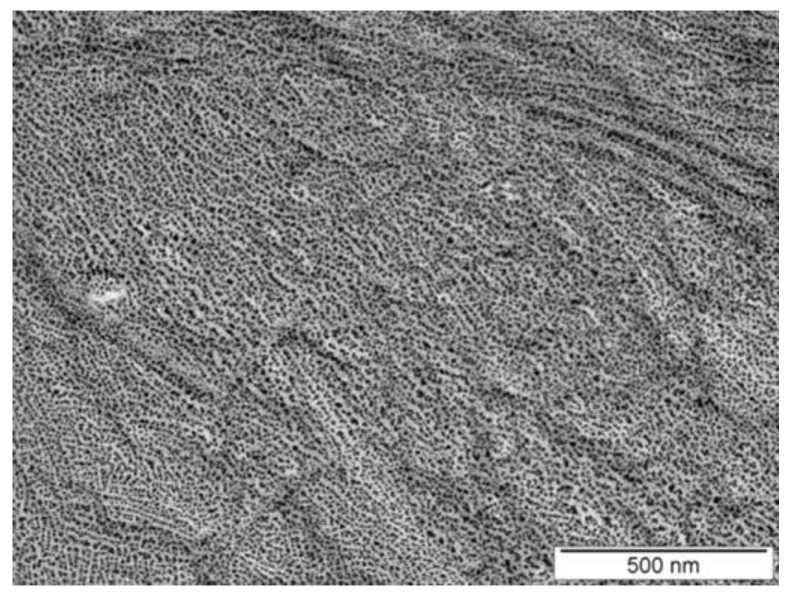
TEM bright-field image of gold-decorated thin films of the sample PE-b-sPP-1 with *f*_sPP_ = 53% slowly crystallized from the melt.

The lamellar morphology suggested by the SAXS data has been confirmed by transmission electron microscopy (TEM). Thin films of the sample PE-*b*-sPP-1 with *f*_sPP_ = 53% were prepared by casting at room temperature on microscope glass slides from a *p*-xylene solution (0.2 wt%–0.5 wt%). The films were melted in a microscope hot stage and crystallized from the melt by slow cooling to room temperature. The TEM bright-field image of the so-crystallized films is reported in Figure 9. The film was coated with gold particles to improve the contrast in the TEM observation and to reveal details of the morphology. The technique of gold decoration is used to visualize edge-on crystalline lamellae of polymers in TEM bright-field images, especially in the case of low-TEM-amplitude contrast between amorphous and crystalline phases [79,80]. The vaporized gold gathers in the ditches made by the interlamellar amorphous material and produces a regular pattern of gold particles, which is observed in bright-field imaging [79,80,81]. In the case of homopolymers, this generally produces thin layers of gold particles at the interface between the amorphous and crystalline lamellae, containing rows of essentially one gold particle thickness [79,80].

In the image of Figure 9 of the sample PE-*b*-sPP-1, the dark spots correspond to the gold particles that are presumably located in the amorphous intra-lamellar phases of PE and sPP, that is, in between the crystalline domains of PE or sPP, whereas the brighter regions correspond to PE and/or sPP crystalline lamellae. The image of Figure 9 has been collected at high magnification on a selected region of spherulites to show the lamellar details. In agreement with the results of the SAXS data, it is apparent from Figure 9 that the PE and sPP crystalline lamellae (the light stripes) alternate with amorphous layers (the darker stripes) and appear locally partially oriented.

## 4. Conclusions

The structure and morphology of crystalline-crystalline block copolymers composed of crystallizable blocks of polyethylene (PE) and syndiotactic polypropylene (sPP) were studied by WAXS and SAXS. Two samples, having different block lengths and sPP volume fractions of 53 and 75%, were analyzed. The samples crystallize in the stable form I of sPP and the orthorhombic form of PE, both in as-prepared and melt-crystallized samples. Both samples show only one broad endothermic peak with shoulders at high temperatures, and only one exothermic peak in DSC thermograms, indicating melting of PE and sPP crystals in the same range of temperature, and almost simultaneous crystallization of PE and sPP during cooling. However, diffraction profiles recorded at different temperatures during cooling from the melt indicate that, in both samples, the PE block crystallizes first at higher temperature (higher than 125 °C), and the sPP block crystallized after at lower temperature.

The morphology of the symmetric sample with volume fraction of sPP of 53% that develops from the melt during cooling upon crystallization of the two blocks, was analyzed by recording SAXS profiles during cooling. The SAXS profile of the melt recorded at 250 °C is essentially featureless and not informative, due to the very small electron density difference between the PE and the sPP blocks in the melt that prevents observation of the eventual phase-separated structure. Upon cooling, three correlation peaks, at values of the scattering vector, *q*_1_ = 0.12 nm^−1^, *q*_2_ = 0.24 nm^−1^ and *q*_3_ = 0.4 nm^−1^, appear in the SAXS profiles, indicating development of a lamellar morphology, where the crystalline lamellae of PE and sPP alternate with amorphous layers. The alternating crystalline domains separated by amorphous layers are made of two different crystalline phases (stacks of sPP and PE lamellae), and each crystalline layer is sandwiched by its own amorphous phase (sPP or PE). This morphology is compatible with both the hypotheses of crystallization from a homogeneous melt and from a segregated melt. In the latter case crystallization would occur confined to the preformed lamellar domains of sPP and PE, giving a global lamellar morphology, where stacks of crystalline lamellae of PE and sPP grow within the corresponding lamellar domains of the two blocks. 

The morphology that developed upon cooling from the melt changes with temperature due to the different contribution of PE and sPP crystallization that occur at different temperatures. At temperatures higher than 120 °C, when only PE crystals are formed and the contribution of sPP crystals is negligible, the SAXS intensity is essentially due to the formation of crystals of PE and the values of the long period of nearly 52 nm and the thicknesses of the amorphous and crystalline layers, refer essentially to stacks of crystalline lamellae of PE (nearly 17 nm thick) alternating with the amorphous layer. At lower temperatures, when crystals of sPP are also well formed the morphology is more complexwhose details, with assignment of the observed periodicities of PE or sPP lamellar stacks, were revealed by analysis of the self-correlation functions of the electron density fluctuations. A model of the morphology at room temperature is proposed based on the correlation distances determined from the self-correlation functions. The lamellar domains of PE (41.5 nm thick) and sPP (8.2 nm thick) alternate, each domain containing stacks of crystalline lamellae sandwiched by their own amorphous phase, forming a global morphology having a total lamellar periodicity of 49.7 nm, characterized by alternating amorphous and crystalline layers, where the crystalline layers are alternately made of PE and sPP lamellae of different thickness. In the crystalline lamellar stacks, the thickness of the PE lamellae is 22 nm, whereas the thickness of the sPP lamellae is only 3.5 nm. This lamellar morphology, with stacks of crystalline lamellae of PE and sPP alternating with amorphous layers, was confirmed by TEM observation.

## Figures and Tables

**Table 1:** Total molecular mass (*M*_n_), molecular masses of PE (*M*_n(PE)_) and sPP (*M*_n(sPP)_) blocks, weight fraction (*w*_PP_) of the sPP block, volume fractions of the sPP (*f*_sPP_) and PE (*f*_PE_) blocks and polydispersity (*M*_w_/*M*_n_) of the samples of PE-b-sPP block copolymers.

Sample	*M*_n_ (Da)	*M*_n(sPP)_ (Da)	*M*_n(PE)_ (Da)	*w*_sPP_ (wt%)	*f*_sPP_ (*v*/*v*%)	*f*_PE_ (*v*/*v*%)	*M*_w_ /*M*_n_
PE-b-sPP-1	20000	10200	9800	51	53	47	1.2
PE-b-sPP-2	64000	46700	17300	73	75	25	1.2

**Table 2:** Values of crystallinity *x*_c_(WAXS) evaluated from the WAXS diffraction profiles of Figure 3B, scattering vectors *q* = 4πsinθ/λ of the three correlation peaks *q*_1_, *q*_2_ and *q*_3_ observed in the SAXS curves of Figure 5 recorded at different temperatures *T* during cooling from the melt of the sample PE-b-sPP-1 with *f*_sPP_ = 53%, values of the corresponding long period (*L*_B_ and *L*_CF_) evaluated from the scattering vector *q*_1_ of the first correlation peak (*L*_B_ = 2π/*q*_1_) and from the self-correlation functions of electron density fluctuations of Figure 7 (*L*_CF_), values of the thicknesses of the crystalline lamellae *l*_c_ and amorphous layers *l*_a_, evaluated from the SAXS profiles of Figure 5B and the values of the long period *L*_B_ (*l*_c_ * and *l*_a_ *) and from the self-correlation functions of Figure 7 (*l*_c(CF)_ and *l*_a(CF)_), and values of the thickness of the PE and sPP domains (*L*_PE_ and *L*_sPP_).

*T* (°C)	*x*_c(WAXS)_ (%)	*q*_1_ (nm^−1^)	*q*_2_ (nm^−1^)	*q*_3_ (nm^−1^)	*L*_B_ = 2π/*q*_1_ (nm)	*l*_c_ * (nm) ^a^	*l*_a_* (nm) ^b^	*L*_CF_ (nm)	*l*_c(CF)_ (nm)	2*l*_a(CF)_ (nm)	*L*_PE_ (nm)	*L*_sPP_ (nm)
130	19	0.125	0.242	0.393	50.3	11.0	39.3	52.0	16.9	35.1		
125	32	0.125	0.242	0.374	50.3	18.1	32.2	49.3	17.3	32.0		
120	37	0.123	0.240	0.40	51.1	21.1	30.0	51.4	16.6 (PE)	34.8		
25	47	0.120	0.241	0.40	52.4	27.0	25.4	49.7	3.5 (sPP) 22 (PE)	4.7 (sPP) 19.5 (PE)	41.5	8.2

(^a^) *l*_c_ * = φ_c_ (WAXS) × *L*_B_, φ_c =_ *x*_c_ *d*_c_^−1^[*x*_c_ *d*_c_^−1^ + (1 − *x*_c_) *d*_a_^−1^] with *d*_c_ = 0.997 g/cm^3^ and *d*_a_ = 0.850 g/cm^3^ the densities of crystals of PE in the orthorhombic form and of the amorphous PE at 298 K, respectively [67]. (^b^) *l*_a_* = *L*_B_ − *l*_c_ *.

## Data Availability

The data in this study are available on reasonable request from the corresponding author.
